# Barriers to Lose Weight from the Perspective of Children with Overweight/Obesity and Their Parents: A Sociocultural Approach

**DOI:** 10.1155/2014/575184

**Published:** 2014-10-02

**Authors:** Ana Lilia Rodríguez-Ventura, Ingris Pelaez-Ballestas, Reyna Sámano-Sámano, Carlos Jimenez-Gutierrez, Carlos Aguilar-Salinas

**Affiliations:** ^1^Departamento de Investigación en Nutrición y Bioprogramación, Instituto Nacional de Perinatología, Piso 2, Torre de Investigación, Montes Urales 800, Colonia Lomas de Virreyes, 11000 Miguel Hidalgo, Mexico City, DF, Mexico; ^2^Departamento de Endocrinología, Hospital Infantil de México Federico Gómez, Mexico City, DF, Mexico; ^3^Departamento de Reumatología, Antropología, Médica Hospital General de México, Mexico City, DF, Mexico; ^4^Departamento de Endocrinología, Instituto Nacional de Ciencias Médicas y de la Nutrición Salvador Zubirán, Mexico City, DF, Mexico

## Abstract

*Introduction.* There are not enough studies about the barriers to lose weight from the perspective of children and their parents.* Methods.* Children and adolescents diagnosed with overweight/obesity in the Department of Endocrinology and their parents were invited to participate in a series of focus group discussions (FGD). Twenty-nine children 10–16 years old and 22 parents participated in 7 focus groups; 2 mothers and 2 adolescents participated in depth interviews. All interviews were recorded, transcribed, and analyzed through grounded theory.* Results.* Parents went to the hospital only when their children presented any obesity complication; for them, overweight was not a health problem. Parents referred to lack of time to supervise about a healthy diet and exercise; besides, the same parents, relatives, friends, and the mass media encourage the consumption of junk food. Children accepted eating a lot, not doing exercise, skipping meals, and not understanding overweight consequences. Both, parents and children, demanded support to do the time recommended for exercise inside the schools. They also suggested getting information from schools and mass media (TV) about overweight consequences, exercise, and healthy food by health workers; they recommended prohibiting announcements about junk food and its sale.* Conclusions.* The barriers detected were lack of perception of being overweight, its identification as a disease and its consequences, lack of time to supervise a healthy lifestyle, and a big social influence to eat junk food.

## 1. Introduction

Rates of severe childhood obesity have tripled in the last 25 years, with significant differences by race, gender, and poverty [1]. In 2006, 70% of adults (30–60 years old) and 35% of adolescents (12–19 years old) were overweight/obese in Mexico [2], but after six years and despite different public health efforts, this high national prevalence continues [3]. A longitudinal study reported only 23% of success to decrease body mass index (BMI) in 83 of 150 adolescents who were followed during 23 months [4]. In a meta-analysis of 64 trials (5230 adolescents), authors concluded that interventions mixing lifestyle and behavior interventions were effective but need to consider psychosocial features to get behavioral changes [5]. The design of studies must consider isolation feelings, understanding of overweight consequences and comorbidities, individual barriers, and the social and cultural context [6–9].

Qualitative research has the goal of explaining the sociocultural world through self-experience of each person acting as theoretical and methodological livelihood; use techniques and designs to get, analyze, and interpret information (narratives, videos, and documents); the results are not expressed numerically [10, 11]. Previous qualitative studies have indicated that parents are not concerned about their children being overweight. Parents have expressed more anxiety about children being underweight than overweight, so then they usually are not aware about the weight status of their children [12, 13]. By the way, children are not aware of their overweight; in fact, a qualitative study found that black female adolescents want losing weight but not too much in order not to be so different to the rest of the family [14]. In addition, parents have referred to the fact that they are depriving their children if they restrict unhealthy food [12]; they are reluctant to restrict 100% fruit juice, need specific strategies to increase vegetable consumption, and think that limiting screen time would be difficult, especially when they are busy or during inclement weather [15]. Other barriers to achieve a healthy lifestyle included cost of healthy food, time and practicality, family preferences, and difficulty in changing habits [16]; individual, family, and community involvement [17].

The aim of this study was to identify the barriers to lose weight, including the weight status perception, beliefs, habits, opinion of social support, and recommendations from the perspective of children being overweight or obese and their parents, in order to improve the interventions of overweight/obesity in children considering this information.

## 2. Methods

### 2.1. Subjects

Participants (children and parents) were recruited from a Pediatric Endocrinology Unit by a pediatrician who contacted them in the waiting area or by phone since a record of children with the diagnosis of overweight or obesity. Eligibility criteria included age (10–18 years old), male or female, overweight (body mass index (BMI) ≥ 85th percentile for age and gender) or obese (BMI ≥ 95th percentile for age and gender), and parents with a child diagnosed with overweight or obesity. The study was approved by the Research and Ethic Committee of Children's Mexico Hospital Federico Gomez and written informed consent was obtained from all parents and children.

### 2.2. Focus Group Methodology

A focus group is a technique of the qualitative method to describe and understand interpretations, perceptions, and beliefs of a group (6–10 persons) with a common problem [18, 19]. The goal is the heterogeneity of the information on persons who share the same problem [11, 18–20]. The theoretical sampling was selected and made according to the wealth of information and not to the number of individuals and the process stops when new more aspects of the same phenomenon are not already mentioned [11].

Focus groups were formed by a moderator-social psychologist, an observant—a physician, and 6–8 children or parents. The observant was a pediatric endocrinologist independent of his/her assigned doctors. There were 4 groups of children, 2 of girls (10–14 and 15–18 years old) and 2 of boys (10–14 and 15–18 years old), and 3 groups of parents, 2 of mothers (younger than 40 years old and older than 40 years) and 1 of fathers (every age). Four interviews were made in depth to complement the information: 2 mothers (28 and 45 years old) and 2 adolescents (girl of 13 years old and boy of 15 years old).

The guide for interview was made by literature review [5–9, 12–15] and by a research team (pediatric endocrinologist, pediatrician, psychologists, nutritionists, and medical anthropologist). The guide explored weight perception, causes of overweight, limitations to lose weight, habits and beliefs, opinions about social support, suggestions to lose weight, and the way to get more information about the health problem. The interviews lasted 90–120 minutes, were recorded, transcribed verbatim, and were run until themes based on parental and children responses achieved saturation. The analysis was made by Atlas.ti, according to grounded theory: identification of important themes, comparison of themes, and organization of each theme in families, codes, or categories [10].

*Example of Coding Scheme for Theme*

Limitations to lose weight are as follows:

- weight status perception,
- weight status perception in children,
- reasons to buy fast food,
- limited time to prepare healthy food,
- limited time to be with children,
- economical limitations,
- poor understanding of healthy diet and habits,
- poor understanding of consequences of overweight.

## 3. Results

### 3.1. Participants

Twenty-nine children and 22 parents participated in this study. The 51.7% were female and the median age was 15 years (10–17 years old). All children presented body mass index (BMI) in percentile 85 or higher. 82% of parents had overweight or obesity and only 22.7% of the children had parent participants in the focus groups. The mothers interviewed in depth were 38 and 45 years old, respectively, the girl 12 and the boy 15 years old.

### 3.2. Themes

**Figure 1:**
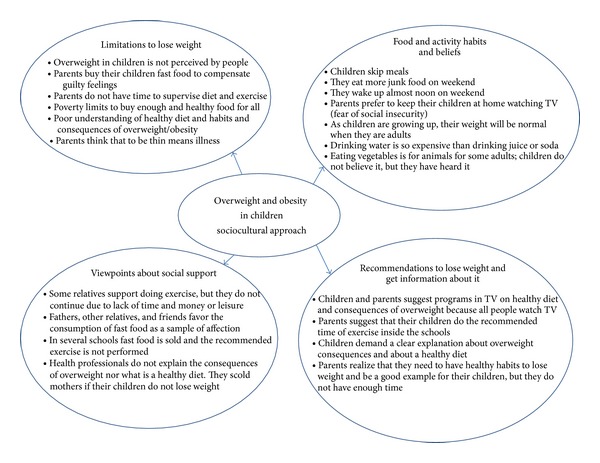
Sociocultural approach about overweight/obesity in children (detected barriers and recommendations from children and parents).

We identify 4 principal themes (Figure 1): limitations to lose weight, eating and activity habits and beliefs, views on the social support, and recommendations for losing weight and disseminate information.

#### 3.2.1. Limitations to Lose Weight

*(1) Lack of Overweight/Obesity Perception. *Almost all parents did not perceive overweight or obesity in their children; they went to the hospital for acanthosis nigricans, hypertension, asthma, or other health conditions but not for the weight of their children:

*I never imagined that she was overweight… when we saw the problem of her skin… consulted a dermatologist and because of the dark neck, told us that she had to go with an endocrinologist…* (Mother, 38 years old).

*(2) Guilt about Abandoning Their Children. *Parents expressed feelings of guilt about leaving their children for a long time for their work and compensating them with a lot of food that they like, videogames, or anything that children demand:

*… His dad thinks that it is giving back to the girl, the fact that it is not a lot of time with her: - Daugther [sic], these are for you, some cookies, small cakes, pizza, Chicken Happy-* (Mother, 42 years old).

*(3) Lack of Time to Supervise the Children*. Mothers do not have time to supervise diet and exercise of their children; some of the mothers offer their children fruits and vegetables but they do not eat them:

*… I cannot leave work… I want to be all the time with her and really be supervising it *(Mother, 42 years old).

*I find the rotting fruit of a very long time…* (Mother, 46 years old).

*(4) Economical Limitations.* The parents do not have enough money to buy fruit or healthier food for each member of the family:

*What concerns me more is the economic part I think that's why I cannot give everyone all the fruits required… it does not reach… are five meals,… three or more fruits and vegetables…* (Mother, 43 years old).

*(5) Lack of Information about Healthier Food and Overweight/Obesity Consequences*. Parents and children did not have an understanding of healthy diet and consequences of overweight because they did not receive a complete explanation about it in the past:

*I had been carrying with several doctors and did not allow him to be a diet so well what it is like now that you say, such a thing is worth so many calories and you can eat as many servings…* (Father interviewed).

#### 3.2.2. Eating and Activity Habits and Beliefs

(*1) Skipping Breakfast or Any Other Time of Food. *Most of the children participants did not have breakfast due to lack of time or appetite, as well as lunch or dinner:

*… I only had time wash up, I did not have time for breakfast and in my school I did not eat, until I were returning home on the night I took dinner* (Female adolescent, 16 years old).

(*2) Insufficient Rest (Short Sleep). *Children go to bed very late because they arrive late at home, do homework, use the computer, play videogames, watch TV, or wait for their parents to come back from their job.

(*3) Disorder on Weekends*. Children and even parents wake up almost at noon, do not respect schedules or quality of their food, do not combine correctly the food groups or their quantities, and usually underestimate the amount of food:

*The* ***only*** *thing that I eat in the evening it is* ***one liter*** *of milk and two loaves of bread* (Adolescent male, 16 years old).

(*4) Lack of Security, Money, and Time to Do Exercise.* For parents it is better that the children are entertained watching TV than in the street exposed to danger; this way, the parents are able to finish work at home. Children mentioned lack of time, nearer places, and money:

*… Is the lack of time… and the other is the insecurity… if you are not with the children is very dangerous, it is the main factor in that the children are locked up* (Father interviewed).

*Comfort for ourselves, if they are entertained watching TV, they are not giving us problems; and we can finish our tasks home…* (Mother, 29 years old).

*I practiced Zumba, arabic dance and hawaiian… but the teachers are no longer going, I was in karate, but by issues of money, I couldn't continue. I was swimming but now for my school I do not have time to go* (Female adolescents, 10–14 years old).

(*5) Practicality and Rejection of the Natural Water.* Several parents said that buying a soda is cheaper and faster than preparing water with fruit. Natural water is unacceptable for several people. “*… my husband tells me that doing water with fruits is more expensive that buying soda… the water, sugar, fruits.” “At home it is a sin eating accompanied with natural water, poor children, how is possible they drink natural water with the food…” *(Focus group of mothers younger than 40 years old).

(*6) Refusal to Eat Vegetables*. The belief “vegetables for animals” was not present in adolescents participants, but some of them have heard it from grandparents, parents, or friends of their parents. “*A friend of mom, was eating and told than the lettuce is for animals” *(Boy, 13 years old).

(*7)* “*Children in Growth Should Not Restrict Their Food.*” Some mothers expressed the idea that children should eat a lot because they are growing. In fact, they expressed that thinness indicates disease and overweight means good health. Adolescents do not share this belief but do not want to be too thin.

*It was normal to listen-Pretty child, he is cute so chubby-…. When children are heavy, we are happy… when people saw my daughter so small and thin, they told me - your child is underweight, she looks sick…* (Father and mother, 42 years old, resp.).

*… I would like to be thin, but not too much* (All male adolescents, 10–14 years old).

#### 3.2.3. Views on the Social Support (Relatives, Friends, etc.)

(*1) Giving Junk Food in Excess as a Display of Affection.* Fathers, friends, and mass media improve excessive junk food consumption as a display of affection.* “… my daughter received a box of chocolate candies when she was operated….” “When we lived in the house of my parents, they told us - How is possible that my grand-daughter does not eat sweets, this is traumatic”  *(Focus group of mothers ≥ 40 years old).

*… because my sons watch toys in the announcement TV of any food and they tell me buy me it or that….. With this type of announcementes [sic] call children, with offerings such as buy 1 and take 2, my son tell me—it is cheap -* (Mothers < 40 years old).

(*2) Poor Support from Schools.* In several schools fast food is sold; in fact, there are no options to eat healthy food.* “… In the school, there is a specific place to sell maruchan soup and it is the first to be sold, fruits and vegetables are not sold, only maruchan soup, cookies, snacks and sweets” *(Female adolescent, 13 years old).

(*3) Lack of Clear Information about Obesity Consequences and Healthy Diet. *Physicians do not explain enough the consequences of obesity and the meaning of a healthy diet. In addition, they scold mothers if their children do not lose weight, although some teens prefer that their doctors speak hardly about consequences in order to understand why they need to change.* “… physicians do not explain us the things… a pediatrician told me the true but very kindly, the other pediatrician told me very serious the true and now I am changing”* (Male adolescent 15 years old).

#### 3.2.4. Recommendations to Lose Weight and Disseminate Information

(*1) Support from TV and Mass Media to Disseminate Information and Regulate Publicity of Junk Food.* Children and parents recommended TV programs about healthy diet and consequences of overweight because all people watch TV.* “… more information in TV because it is the principal mass media, in TV, the experts must explain about diseases caused by the overweight…”* (Mother, 38 years old).

(*2) Real Support from Schools.* Parents considered that the school must also educate children about healthy lifestyle because children stay there most of their time. Children also were interested to learn about healthy food and overweight consequences in the school, by health workers. They recommended prohibiting the selling of fast and junk food and placing dining rooms offering healthy food. They also suggested that children do exercise on recommended time inside schools:* “The actions should be directed in the schools because it is where children are the greatest time…” *(Focus group of fathers).

(*3) Example from Parents.* Parents realize they must practice being a good example.* “I feel that if I lose weight, it would be the best motivation for my son” *(Mother, 36 years old).

## 4. Discussion

The lack of perception of overweight/obesity and of its condition as a disease with comorbidities favors the poor adherence to treatment [21]. In Canada, some authors reported that more than 44% underestimated children body size and also 33% of their physician [22]. Between 32.1 and 87.5% of mothers perceive the weight of their children who present overweight or obesity as normal [23–25]. The parents of this study accepted that they themselves, relatives, and friends believed that being thin means disease or debility; it is the reason why everyone recommends eating a lot to gain weight if the children were thin. Despite the fact that children participants have listened to this idea from some adults, they said they did not agree; however, they expressed wanting to lose weight but not too much although their weight was excessive. It is also possible that Mexican children do not want to lose a lot of weight in order not to be so different to the rest of the family, same as a qualitative study reported it [14] if we consider that 70% of adults in Mexico are overweight or obese. If the health personnel do not identify this lack of perception of overweight and its comorbidities, the interventions will continue without success. Mexico, until 1988, had a very high prevalence of underweight in children, so then it is also possible that people prefer to see children overweight than underweight. This low perception of being overweight and knowledge about its consequences were also recently reported in USA [26].

On the other hand, the principal limitation referred by parents was the lack of time to supervise their children; several studies have reported that the active family participation (principally parents) encourages the change toward healthy habits in children [27, 28]. In fact, in people with diabetes, family support is frequently recognized as an important factor in lifestyle changes, but only 13% of the respondents with diabetes reported that their families had made any adjustments to their lifestyles that would benefit them [29]. The same parents participants realize that if they lose weight, their children will also lose weight, as Braet has referred about the importance of telling the children “do as I do” in place of saying “do as I say” [30].

Another important barrier is the lack of understanding about overweight consequences. In fact, all parents and children participants, especially, the oldest, demanded a clear explanation about it. This clear explanation would be the best strategy to motivate parents and children to lose weight [25] and to forget the belief in adults about association between underweight and disease or overweight and good health. In a previous study, authors found that one of the reasons why children and adolescents would participate in a program to lose weight is to prevent diabetes [26]. Bolling et al. [15] interviewed parents of preschool children with overweight/obesity and they expressed, such as our participants, their interest to understand clearly the health risks being overweight and obese because it is not easy to discuss with children the importance of eating vegetables and watch TV for less time.

Parents feel guilty leaving their children alone for their jobs and, as compensation, they buy them junk food. In addition, this sense of guilt also limits their authority even more if the same parents are unable to follow a healthy lifestyle [28, 30]. Parents reported that children got angry when they were restricted to eat certain foods or demanded to do exercise [31]; one of the participating mothers said that she and her daughter consult a psychologist to improve their relationship; they have serious differences regarding food. In fact, recently, in a qualitative study of Mexican-American and Mexican immigrant, the majority of parents described being permissive and allowing unhealthy food choices [32]. Usually, when people are imposed to change, without the freedom to make their own choices, this makes it totally the opposite, so it is important to make sure that the child wants to change and is willing to do so [33].

Several authors have reported, from the perspective of physicians interviewed, that the therapeutic success is low because patients and relatives do not have motivation, there is no family support, the mass media influence their elections of food, and there are no brochures more comprehensive and practical on healthy eating and exercise [34–37], and just this impression was also shared by the participants (children and parents) of this study.

Interventions in secondary schools have improved the sale of food [31, 38–40], which also suggested participants in our study, in addition to install dining rooms that offer healthy and balanced meal. In fact, in the State of Mexico, two communities with similar sociodemographic characteristics were randomized to implement an intervention (*n* = 816) or serve as a control (*n* = 408). The intervention was carried out in primary schools and it consisted of education on healthy habits, modification of distributed food, and physical activity. Until after three years, intervention resulted in a lower increase of BMI (1.6 versus 1.9 Kg/m^2^,  *P* < 0.01) and a decreased consumption of total calories, bread, fat, and sugar consumption in the schools [41]. On the other hand, as the school is the place where children are spending a great number of hours, it is the best place to play and do physical activity with trained professionals in order to increase the playtime of children, as our participants suggested in the focus groups [42]. In fact, in Israel, Stein et al. recently published that the psychosocial mediators include the influence of the family and peer environment and exposure to the media and our participants also mentioned the big influence of TV, so then this author concluded that prevention programs should be multidisciplinary, combining the knowledge of experts from different professions and taking into consideration the important role of the family environment and relevant influential social organizations, particularly school [43].

The principal limitation of this study was at the same time a stronghold because our participants were already alive to the problem. In the past, they did not perceive the overweight in their children, did not identify it as a disease, and ignored the consequences and the best way to eat; now, they had more knowledge to give recommendations in order to decrease the weight. However, the perspective could be a little different, interviewing people without the perception of overweight/obesity in children and their parents.

In conclusion, the barriers detected in this study have similitude and differences with other big cities. The intervention programs must consider the lack of perception of being overweight/obese, its identification as a disease, and its consequences; the lack of time of parents to supervise diet and exercise of their children; the great influence of relatives, friends, school, and mass media to eat junk food and the possibility to educate about it from schools and mass media (principally TV) by health personnel.
